# Outlook for tuberculosis elimination in California: An individual-based stochastic model

**DOI:** 10.1371/journal.pone.0214532

**Published:** 2019-04-09

**Authors:** Alex J. Goodell, Priya B. Shete, Rick Vreman, Devon McCabe, Travis C. Porco, Pennan M. Barry, Jennifer Flood, Suzanne M. Marks, Andrew Hill, Adithya Cattamanchi, James G. Kahn

**Affiliations:** 1 Stanford University School of Medicine, Palo Alto, CA, United States of America; 2 Consortium to Assess Prevention Economics (CAPE), University of California San Francisco, San Francisco, CA, United States of America; 3 Division of Pulmonary and Critical Care Medicine, University of California San Francisco, San Francisco, CA, United States of America; 4 Curry International Tuberculosis Center, University of California, San Francisco, San Francisco, CA, United States of America; 5 Proctor Foundation, University of California San Francisco, San Francisco, CA, United States of America; 6 Department of Epidemiology and Biostatistics, University of California San Francisco, San Francisco, CA, United States of America; 7 Tuberculosis Control Branch, California Department of Public Health, Richmond, CA, United States of America; 8 Division of Tuberculosis Elimination, National Center for HIV, Hepatitis, STI, and TB Prevention, Centers for Disease Control and Prevention, Atlanta, GA, United States of America; 9 Philip R Lee Institute for Health Policy Studies, University of California San Francisco, San Francisco, CA, United States of America; University of Cape Town, SOUTH AFRICA

## Abstract

**Rationale:**

As part of the End TB Strategy, the World Health Organization calls for low-tuberculosis (TB) incidence settings to achieve pre-elimination (<10 cases per million) and elimination (<1 case per million) by 2035 and 2050, respectively. These targets require testing and treatment for latent tuberculosis infection (LTBI).

**Objectives:**

To estimate the ability and costs of testing and treatment for LTBI to reach pre-elimination and elimination targets in California.

**Methods:**

We created an individual-based epidemic model of TB, calibrated to historical cases. We evaluated the effects of increased testing (QuantiFERON-TB Gold) and treatment (three months of isoniazid and rifapentine). We analyzed four test and treat targeting strategies: (1) individuals with medical risk factors (MRF), (2) non-USB, (3) both non-USB and MRF, and (4) all Californians. For each strategy, we estimated the effects of increasing test and treat by a factor of 2, 4, or 10 from the base case. We estimated the number of TB cases occurring and prevented, and net and incremental costs from 2017 to 2065 in 2015 U.S. dollars. Efficacy, costs, adverse events, and treatment dropout were estimated from published data. We estimated the cost per case averted and per quality-adjusted life year (QALY) gained.

**Measurements and main results:**

In the base case, 106,000 TB cases are predicted to 2065. Pre-elimination was achieved by 2065 in three scenarios: a 10-fold increase in the non-USB and persons with MRF (by 2052), and 4- or 10-fold increase in all Californians (by 2058 and 2035, respectively). TB elimination was not achieved by any intervention scenario. The most aggressive strategy, 10-fold in all Californians, achieved a case rate of 8 (95% UI 4–16) per million by 2050. Of scenarios that reached pre-elimination, the incremental net cost was $20 billion (non-USB and MRF) to $48 billion. These had an incremental cost per QALY of $657,000 to $3.1 million. A more efficient but somewhat less effective single-lifetime test strategy reached as low as $80,000 per QALY.

**Conclusions:**

Substantial gains can be made in TB control in coming years by scaling-up current testing and treatment in non-USB and those with medical risks.

## Introduction

California has the largest share of tuberculosis (TB) cases among U.S. states, composing 22% of the national TB burden in 2015 [1]. For the United States and other low incidence settings, the World Health Organization (WHO) in 2014 recommended that a pre-elimination target (less than 10 cases per million population) be met by 2035 and elimination (less than 1 case per million) be reached by 2050 [2]. California has made great strides towards achieving these goals; the number of cases of active TB has decreased from 5,150 in 1993 to 2,133 in 2015, and the rate has fallen from 164 to 55 annual cases per million population [3]. These dramatic reductions in California were due in part to screening of recent immigrants, decreasing TB transmission, aggressive case finding, increasing treatment of HIV, and favorable demographic changes [4]. Yet, progress has slowed. From 1993–1997, there was a 6.4% annual decline in cases compared to a decline of 3.8% during 2005–2014 [3].

Although the exact reasons for this slowed decline in California are unclear, the trend is likely due to a rising proportion of TB incidence due to reactivated latent tuberculosis infection (LTBI) in an established non-US-born (non-USB) population of 11 million. Of the 2,133 cases in 2015, 79% were attributed to reactivation of remote LTBI, 14% were attributed to recent transmission within California (based on genotyping data from 2013–2015), and 7% occurred in among persons within one year of arrival to the United States (likely representing recent infection outside California or arrival with active disease). Most of the TB cases, 1,773 (83%), occurred in non-USB persons [3].

As reactivation from LTBI becomes an even greater driver of TB in California, risk factors for progression from latent to active disease will play an important role in the future course of the disease. Behavior and conditions such as cigarette smoking, diabetes, HIV, end-stage renal disease (ESRD), and use of tumor necrosis factor (TNF)-alpha inhibitor medications or post-transplant immunosuppressive drugs have been found to increase an individual’s risk of reactivation by two to 25-fold [5]. An estimated 8.7 million individuals have these risk factors, and accounted for 37% of California TB cases in 2015 [4].

It is widely accepted that to reach elimination, California, like other low-incidence settings, needs to conduct targeted testing and treatment (TTT) for those with LTBI. Past models of TB in the United States suggest that preventative therapy for those with LTBI can play a crucial role in reducing TB incidence. [6] Conducting TTT in particular at-risk populations rather than in the general population might offer a more cost-effective option. In this study, we use a novel micro-simulation model to evaluate the costs and effects of potential TTT strategies to reach elimination in California.

## Methods

### Model development

We developed a discrete-time stochastic microsimulation model of 30,000 individuals, representing at a 1:1000 ratio the population of California over age fourteen from 2001 to 2065. Individuals entered the model by maturing-in at 15 years of age or through immigration; they exited the model through death or migration out of California. California-specific demographic details for entrants, such as sex, age group, race/ethnicity, and birthplace were obtained from the American Community Survey [7]. Healthcare workers required to have annual TB tests (4.6% of the population) were modeled as a distinct group. A full explanation of the model structure and input data is available in S1 File.

The natural history of TB was simulated with a Markov chain that separated LTBI into recent (<3 years) and remote infections. Prevalence of LTBI was estimated from the national-level 2011–2012 NHANES data.[8] Adjustments to the LTBI prevalence by race, age, and country of birth were made using California-level demographic data derived from the American Community Survey and estimates of prevalence in foreign nations, as (S1 File) [7–9]. We estimated that LTBI progressed to active disease at a base rate of 73 per 100,000 person-years [10]. The rate was elevated in the presence of a recent infection, a medical risk factor, or both in combination. The model captured six medical risk factors: cigarette smoking, diabetes, HIV, ESRD, and use of TNF-alpha inhibitor or post-transplant immunosuppressive drugs. Each risk factor was distributed into the population in accordance with demographic characteristics and carried a higher baseline mortality rate compared to those without risk factors as described (S1 File). Transmission of *Mycobacterium tuberculosis* was included using a semi-random mixing model that assumed an increased likelihood of interaction with individuals of same race-nativity group.

**Fig 1:**
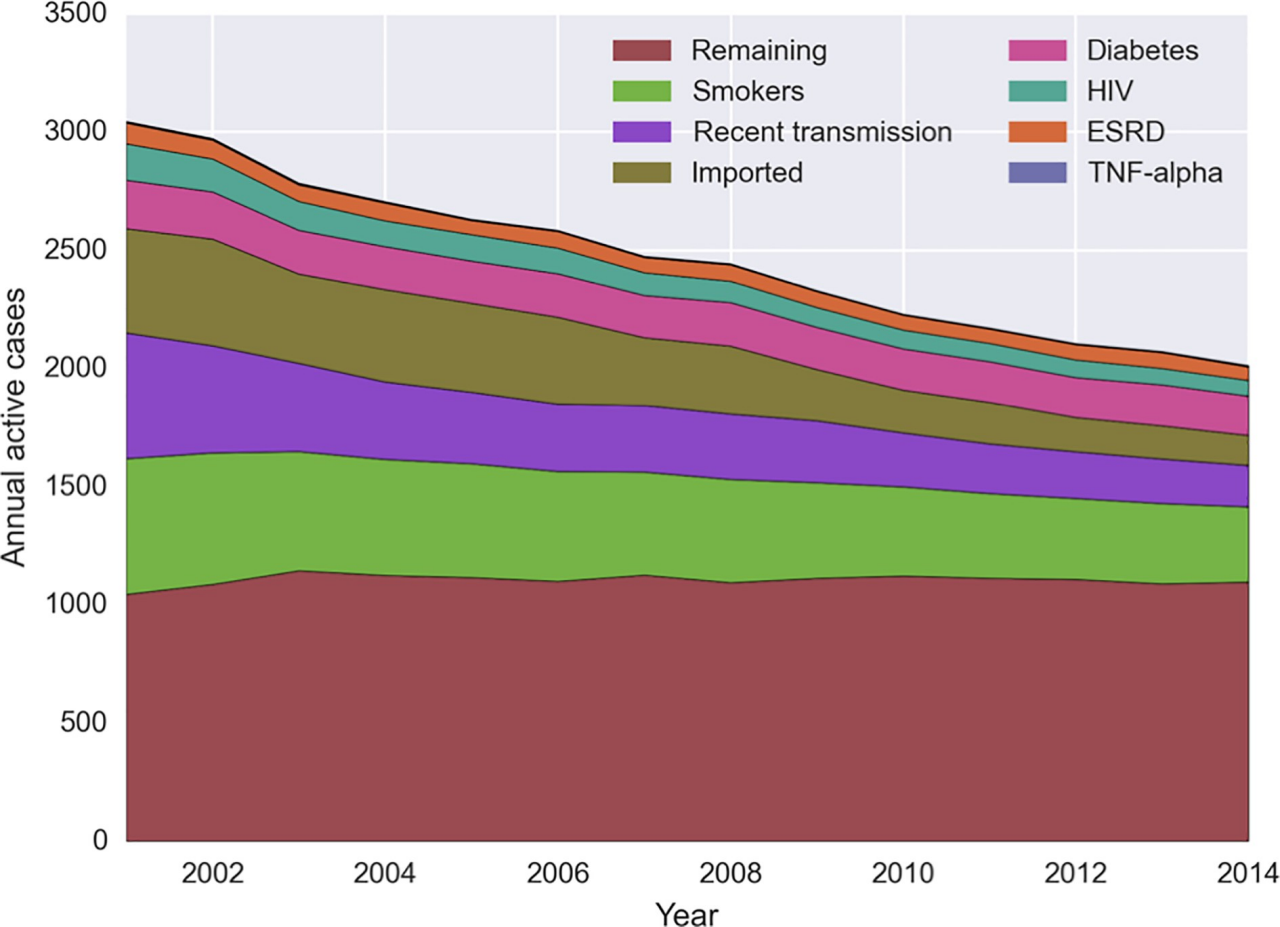
Modelled cases attributable to medical risk factors, recent transmission, and imported cases, 2001–2014. In 2014, the major risk factors for TB were smoking (267 attributable cases), diabetes (162), ESRD (74), HIV (51), transplant (3) and TNF-alpha (1). Increased risk due to recent transmission accounted to 146 cases, and 80 cases were imported. The remaining 1096 cases had no identified risk factor.

**Fig 2:**
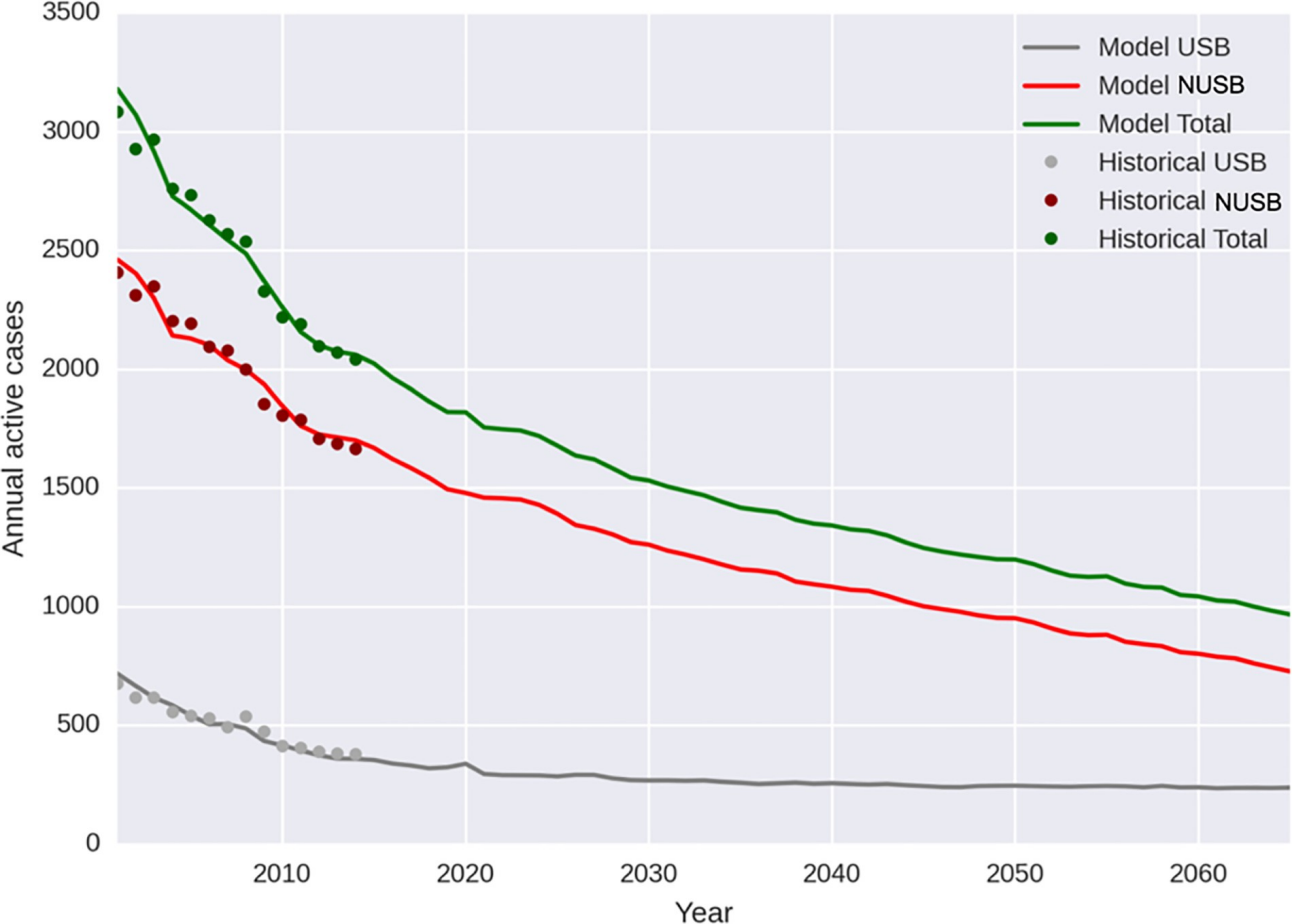
Results of calibration. Mean number of TB cases predicted by model (250 iterations) and historic TB case data reported to California Department of Public Health, in the US-born (USB) and non-USB (NUSB), 2001–2014. For uncertainty ranges in modeled predictions, see Fig 3.

To calibrate the model, we first matched population sizes, and medical risk factor prevalence to California historical data from 2001 to 2014, then applied emigration rates, race and nativity-specific risks of reactivation, and TB incidence risks associated with medical risk factors. To align with the decline in TB cases between 2001 and 2014 (Fig 1 and Fig 2), we manually varied the risk of imported TB disease and of within-CA TB transmission over this time period based on evidence suggesting evolving trends in these factors (S1 File).

### Intervention strategies to aim for elimination

We created a base case scenario that represented current TTT, which include annual testing of healthcare workers and 2% of the population (chosen at random) [7, 11]. The base case scenario assumed a 1:1 mixture of testing with tuberculin skin tests (TST) or QuantiFERON-TB Gold (QFT) interferon gamma release assays as well as LTBI therapy of six months of self-administered isoniazid (INH) for those testing positive. Six months of INH, although not the national recommendation, is the minimum duration of therapy recommended by agencies in California and was assumed to represent the most realistic current practice [12–13]. Costs (in 2015 dollars), sensitivity, and specificity of the tests were estimated from empirical data (S1 File).

We assessed the effect of scaling-up TTT, assuming testing with QFT (a more sensitive and specific test) and treatment with three months of weekly directly-observed isoniazid and rifapentine (3HP) with its higher completion rates (82% of those beginning therapy complete compared to 69% in 6H alone as reported by one trial) [14]. We analyzed four intervention strategies: (1) increased TTT in individuals with medical risk factors regardless of nativity, (2) increased TTT in all non-USB, (3) increased TTT in all non-USB and those with medical risk factors regardless of nativity, and (4) universal testing and treatment in all Californians. For each strategy, we estimated the effects of increasing TTT by a factor of 2, 4, or 10 from the base case scenario. This created a total of twelve simulations in addition to the base case. These strategies permitted re-testing of individuals, to catch those missed due to imperfect test sensitivity, lack of treatment, or reinfection. We also conducted a sensitivity analysis for all 2-fold strategies that tested individuals only once per lifetime.

For each intervention strategy, we estimated the number of TB cases reported, incremental cases prevented with each strategy, and net and incremental costs from 2017 to 2065 in 2015 U.S. dollars using multiple runs of the model. Individuals living in 2065 were then modeled to their death based on estimated lifespan (S1 File). Efficacy, healthcare system costs, adverse events, and dropout rates for each treatment were estimated from available data (Table 1). We estimated the cost per additional case averted and per additional quality-adjusted life year (QALY) gained [15]. We assumed that each case averted confers 1.7 QALYs for those without medical risk factors and 1.1 QALYs, on average, for those with medical risk factors (Table 1). Future costs and QALYs were discounted to the present at 3% (95% confidence interval of 1–5%) per year. Uncertainty was characterized using probabilistic analyses based on probable ranges of values (Table 1).

**Table 1:** Estimated values for single input parameters.

Name	Base	Low	High	Reference
**TB characteristics**				
Number of LTBI cases caused by one active case (transmission coefficient)	4.00	3.95	15.00	Calibration, [31]
Base risk of progression, monthly	0.0000609	0.00	0.00	Calibration, [32]
Proportion of LTBI treated	0.165	0.05	0.2	[33]
Fast latent progression	0.0008097	0.00	0.00	Derived, [24]
**Test characteristics**				
Proportion enroll post + TST LTBI test	0.76	0.72	0.80	[34, 35]
Proportion enroll post +QFT	0.83	0.76	0.89	[34, 35]
Non-USB TST Sensitivity	0.83	0.71	0.87	S1 File
Non-USB TST Specificity	0.82	0.47	0.92	S1 File
Non-USB QFT Sensitivity	0.85	0.70	0.97	S1 File
Non-USB QFT Specificity	0.99	0.98	1.00	S1 File
**Proportion of started who complete treatment**				
6H	0.63	0.54	0.71	[36, 37]
3HP	0.82	0.62	1.00	[14]
**Costs**				
TST	9.51	8.37	10.59	[38]
QFT	84.35	74.00	105.44	[39]
Cost of active TB case (California)	31400	13377	39250	[40]
6H	431.47	323.60	539.34	[20, 21]
3HP	840.64	630.48	1050.80	[20, 21]
**Efficacy of therapy**				
9H	0.92	0.90	0.93	[41, 42]
6H	0.69	0.52	0.86	[41, 42]
3HP	0.92	0.69	1.00	Set equal to 9H
**TST positivity prevalence (%)**				
Non-USB	19.4	16.1	25.8	[8]
US-born	2.4	0.9	2.6	[8]
**Prevalence of risk factors (%), 2014**				
Diabetes	8.9	8	10	[43, 44]
Smoking	12.7	9.5	16	[43, 44]
HIV	0.41	0.3	0.5	[45]
TNF-alpha	0.80	0.5	1	Derived from [46–51]
Solid-organ transplants	0.17	0.15	0.19	[52]
ESRD	0.35	0.21	0.46	[53]
**Relative risk of reactivation for risk factors**				
Diabetes	1.6	1.3	3.6	S1 File
Smoking	2.5	1	4	S1 File
HIV	5.4	2.9	22	S1 File
TNF-alpha	4.7	2.5	5.3	S1 File
Solid-organ transplants	2.4	1.7	18	S1 File
ESRD	11	2	20	S1 File
**Health outcomes**				
QALYs lost per TB case (no medical risk factor)	1.7	1.3	2.6	Calculated, [15]
QALYs lost per TB case (medical risk factors)	1.1	0.83	1.7	Calculated, [15]
**Adverse effects**				
Risk of mild hepatitis, any regimen	0.0075	0.005	0.01	[54]
Risk of severe hepatitis, any regimen	0.002	0.001	0.003	[54]

See S1 File for additional inputs. 9H was not used in analysis, but only included in table for comparison.

### Intervention effect simulation

To compare intervention strategies to the base case, we ran the model for 250 “sets,” defined as one simulation iteration of the base case and of each relevant intervention strategy. Within each set, we removed randomness: we fixed input parameter values (using Monte Carlo sampling) and the random number sequence used to determine each simulation event. This allows us to more precisely quantify how intervention strategies affect outcomes across different but stable epidemic environments.

**Fig 3:**
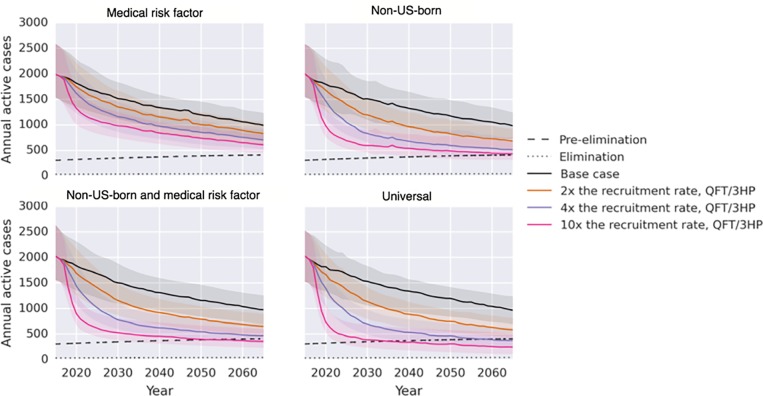
Annual cases of TB projected for 2017–2065, for LTBI test and treat strategies targeted to those with medical risk factors, the non-USB, both of these groups, and all (universal), for 2, 4, and 10-fold increases in testing rates. Dotted and dashed lines show elimination and pre-elimination targets. Solid lines represent mean cases from 250 iterations using best available estimates. Shaded areas represent 95% confidence interval from 250 iterations using probabilistic sensitivity analysis.

Acceptable convergence of the 250-iteration sets is evident in results exhibits. In Fig 3, the base case mean lines are very similar across the four intervention comparisons (each representing 250 sets). In Table 2, the 95% confidence intervals are relatively narrow (3-fold range or less) for case, cost, and cost-effectiveness increments.

**Table 2:** Major results of TB targeted testing and treatment strategies by target population, 2017–2065, main analysis [2].

		Active cases occurring	Cases averted compared to baseline	Incremental cases averted (compared to prior strategy)	Net cost (undiscounted)	Net costs (discounted)	Incremental costs (discounted)	Cost per case averted (ICER^1^), both discounted
**Base-case**		106k (72k - 144k)	N/A	N/A	12b (11b - 14b)	5.9b (5.4b - 6.6b)	N/A	N/A
**Non-USB**	**2x**	79k (53k - 108k)	27k (11k - 37k)	27k (11k - 37k)	15b (14b - 16b)	7.8b (7.1b - 8.3b)	1.9b (1.7b - 2.0b)	192k (136k - 384k)
	**4x**	60k (41k - 87k)	45k (18k - 66k)	19k (5k - 28k)	21b (20b - 22b)	11b (10b - 11b)	3.2b (2.9b - 3.3b)	394k (254k - 930k)
	**10x**	48k (33k - 74k)	58k (29k - 85k)	13k (7k - 19k)	36b (34b - 38b)	19b (18b - 19b)	7.9b (7.0b - 8.6b)	1.2m (662k - 2.1m)
**Medical risk factor**	**2x**	93k (63k - 130k)	13k (5k - 20k)	13k (5k - 20k)	14b (13b - 16b)	7.1b (6.6b - 7.7b)	1.2b (1.0b - 1.3b)	249k (176k - 628k)
	**4x**	81k (56k - 116k)	24k (10k - 31k)	11k (5k - 17k)	19b (18b - 20b)	9.4b (8.9b - 10.0b)	2.3b (2.1b - 2.5b)	485k (333k - 1.0m)
	**10x**	71k (50k - 106k)	35k (17k - 51k)	10k (5k - 23k)	33b (31b - 36b)	17b (15b - 18b)	7.3b (6.4b - 8.1b)	1.5m (788k - 2.9m)
**Non-US born + Medical risk factor**	**2x**	77k (54k - 107k)	29k (13k - 38k)	29k (13k - 38k)	17b (16b - 18b)	8.6b (7.9b - 9.2b)	2.7b (2.5b - 2.8b)	255k (182k - 479k)
	**4x**	56k (39k - 85k)	50k (25k - 64k)	21k (12k - 30k)	26b (24b - 27b)	13b (12b - 14b)	4.8b (4.4b - 4.9b)	531k (375k - 940k)
	**10x**	41k (26k - 73k)	64k (32k - 82k)	14k (7k - 20k)	50b (47b - 52b)	26b (24b - 27b)	13b (11b - 14b)	1.7m (1.1m - 3.2m)
**Universal**	**2x**	72k (43k - 100k)	34k (16k - 43k)	34k (16k - 43k)	22b (20b - 24b)	11b (10b - 12b)	5.5b (5.3b - 5.8b)	455k (356k - 911k)
	**4x**	48k (28k - 80k)	58k (29k - 72k)	25k (13k - 34k)	42b (40b - 44b)	22b (21b - 23b)	10b (10b - 11b)	1.0m (777k - 1.9m)
	**10x**	30k (19k - 65k)	76k (40k - 94k)	18k (10k - 23k)	106b (101b - 115b)	54b (52b - 58b)	33b (30b - 37b)	3.6m (2.6m - 7.3m)

## Results

### Cases

Our base case model estimates that in the 2017–2065 period, there will be approximately 106,000 cases of active TB in California, with a 95% confidence interval of 72,000–144,000 derived from probabilistic analysis (Table 2). Half of these cases will occur before 2037. Projected cases under different scenarios are shown in Fig 3.

Scaling-up TTT in those who were non-USB, from 2017–2065 by 2-fold, 4-fold, and 10-fold was associated with averting 27,000 (95% uncertainty intervals reported in tables), 19,000, and 13,000 additional cases compared to the prior strategy, respectively, suggesting diminishing returns to added TTT (Table 2).

Increasing TTT in those with medical risk factors results in fewer TB cases averted than scaling up TTT in the non-USB, due to both less testing (smaller population) and lower LTBI prevalence (6% in those with medical risk factors vs. 19% in non-USB). The 2-fold, 4-fold, and 10-fold strategies in those with medical risk factors was associated with averting 13,000, 11,000, and 10,000 cases compared to the prior strategy, respectively (Table 2). Increasing TTT in both non-USB and those with medical risk factors produced a slightly higher reduction in TB than increased TTT in the non-USB alone by capturing US-born individuals with medical risk factors. The addition of those with medical risk factors increased the cases averted by 9% on average, compared to increasing TTT in the non-USB alone.

The relationship between increased TTT and number of cases averted is not linear. When individuals with LTBI were identified and treated, they were removed from the pool of persons who undergo testing. Entrants into the model with LTBI were fewer. Thus, the chance of a tested individual having LTBI fell over time, especially with more aggressive TTT. In the 10-fold scale-up for individuals with medical risk factors, the number of tests to successfully treat one case of LTBI rose from 52 in 2017 to 1400 in 2065.

Pre-elimination was achieved by 2065 in three scenarios using best-estimate inputs (Fig 3): a 10-fold increase in the non-USB and persons with medical risk factors (by 2052), and 4- or 10-fold TTT increase in all Californians (by 2058 and 2035, respectively). TB elimination was not met by any iterations of the modeled scenarios, which ended in 2065. The most aggressive strategy, 10-fold in all Californians, achieved a case rate of 8 (95% UI 4–16) active cases per million by 2050.

In the sensitivity analysis, we assessed all 2-fold strategies but limited individuals to a single lifetime test. A similar number of total cases were averted in these strategies compared to the main analysis where re-testing was permitted. For example, in the strategy with 2-fold higher testing of non-USB, 25,000 cases were averted compared to the base case, similar to 27,000 in the main analysis. Cases averted compared to base case for the 2-fold increase in those with medical risk factors, combined non-USB and those with medical risk factors, and the full population were 13,000, 26,000, and 26,000, respectively. However, in the sensitivity analysis, more of these cases were averted earlier in the simulation, as more unique individuals were tested earlier. For example, a 2-fold increase in once-in-lifetime testing in the non-USB achieved a case rate of 1080 cases per year by 2030, compared with 1300 cases per year for the main analysis. As the simulation progresses, the number of cases in the once-in-lifetime approach flattens, since the strategy eventually tests all eligible individuals. For example, the 2-fold universal strategy with no re-testing reaches only as low as 21 cases per million by 2050. Thus, pre-elimination was not achieved by any of the once-in-lifetime approaches.

### Costs

The model estimates that at current trends, testing and treatment of TB from 2017 through 2065 will cost $12.0 billion, discounted to $5.9 billion in 2017 (Table 2). Of this discounted total, more than half ($3.9 billion) is incurred through testing individuals at risk for latent or active disease. Treatment of active disease will cost $1.4 billion, while treatment of LTBI will cost $0.6 billion. Increasing the uptake of TTT resulted in higher testing and LTBI treatment costs and a reduction in active TB disease costs. For example, doubling the TTT rate in the non-USB resulted in $7.8 billion in discounted costs, with 34% and 126% increases in testing and LTBI treatment costs, respectively, and a 21% reduction in active disease cost.

Of scenarios that reached pre-elimination by 2065, each incurred an additional discounted net cost of between $20 and $48 billion compared to the base-case estimates. The least costly scenario that met pre-elimination by 2065 was to test and treat non-USB Californians and those with medical risk factors at 10-fold the current rate; this was estimated to achieve pre-elimination by 2052 at a discounted cost of $20 billion, compared to the base-case. Considering a budgeting perspective, the Year 1 cost for the base case is $217 million, and the least expensive strategy to reach pre-elimination is 4-fold TTT in all Californians, at $847 million (S1 File).

In the one-time testing sensitivity analysis, costs were improved compared to the main analysis, given the fewer tests administered in total. The total discounted cost of TTT was $6.8 billion (95% UI $6.4 billion -$7.4 billion), $7.2 billion (95% UI $6.6 billion—$7.8 billion), $7.9 billion (95% UI $7.5 billion—$8.3 billion) and $9.8 billion (95% UI $9.4 billion—$10 billion) for the 2-fold increase in TTT in those with medical risk factors, the non-USB, those with medical risk factors and the non-USB, and the general population, respectively.

### Cost-effectiveness

Compared with the base case, none of the scenarios in the main analysis fell below $100,000 per additional QALY gained, a commonly cited willingness-to-pay incremental cost-effectiveness ratio (ICER) [16]. Cost-effectiveness varied from a $167,000 per QALY compared to the base-case for the 2-fold TTT increase in the non-USB to a $1.4 million per QALY compared to the base-case in the 10-fold TTT increase in the general population (Table 3).

**Table 3:** Cost-utility results of TB targeted testing and treatment strategies by target population, 2017–2065. costs in 2015 dollars.

			Active cases occurring	Cases averted compared to baseline	Net costs (discounted)	Incremental costs (discounted)	QALYs gained vs prior option	Cost per QALY gained (ICER^2^), CA-only	Cost per QALY gained (ICER^2^), CA and non-CA
**Main results–allows retesting individuals**	**Base-case**		106k (72k - 144k)	N/A	5.9b (5.4b - 6.6b)	N/A	N/A	N/A	N/A
	**Non-US born**	**2x**	79k (53k - 108k)	27k (11k - 37k)	7.8b (7.1b - 8.3b)	1.9b (1.7b - 2.0b)	11k (5.4k - 16k)	167k (116k - 347k)	118k (84k - 210k)
		**4x**	60k (41k - 87k)	45k (18k - 66k)	11b (10b - 11b)	3.2b (2.9b - 3.3b)	9.4k (4.1k - 14k)	339k (210k - 785k)	246k (159k - 470k)
		**10x**	48k (33k - 74k)	58k (29k - 85k)	19b (18b - 19b)	7.9b (7.0b - 8.6b)	7.3k (3.5k - 12k)	1.1m (581k - 2.4m)	803k (463k - 1.6m)
	**Medical risk factor**	**2x**	93k (63k - 130k)	13k (5k - 20k)	7.1b (6.6b - 7.7b)	1.2b (1.0b - 1.3b)	4.7k (1.5k - 6.0k)	258k (183k - 780k)	204k (146k - 518k)
		**4x**	81k (56k - 116k)	24k (10k - 31k)	9.4b (8.9b - 10.0b)	2.3b (2.1b - 2.5b)	4.6k (1.7k - 6.9k)	519k (318k - 1.4m)	414k (277k - 953k)
		**10x**	71k (50k - 106k)	35k (17k - 51k)	17b (15b - 18b)	7.3b (6.4b - 8.1b)	3.8k (1.1k - 7.3k)	2.0m (944k - 7.0m)	1.5m (794k - 3.9m)
	**Non-US born + medical risk factor**	**2x**	77k (54k - 107k)	29k (13k - 38k)	8.6b (7.9b - 9.2b)	2.7b (2.5b - 2.8b)	12k (5.6k - 16k)	226k (159k - 455k)	162k (118k - 293k)
		**4x**	56k (39k - 85k)	50k (25k - 64k)	13b (12b - 14b)	4.8b (4.4b - 4.9b)	10k (5.1k - 14k)	477k (315k - 916k)	346k (239k - 583k)
		**10x**	41k (26k - 73k)	64k (32k - 82k)	26b (24b - 27b)	13b (11b - 14b)	7.1k (2.6k - 11k)	1.8m (1.0m - 4.9m)	1.3m (800k - 2.6m)
	**Universal**	**2x**	72k (43k - 100k)	34k (16k - 43k)	11b (10b - 12b)	5.5b (5.3b - 5.8b)	14k (6.0k - 17k)	408k (314k - 946k)	287k (227k - 539k)
		**4x**	48k (28k - 80k)	58k (29k - 72k)	22b (21b - 23b)	10b (10b - 11b)	11k (4.5k - 14k)	936k (704k - 2.4m)	657k (516k - 1.3m)
		**10x**	30k (19k - 65k)	76k (40k - 94k)	54b (52b - 58b)	33b (30b - 37b)	6.7k (3.4k - 11k)	4.9m (3.1m - 31.4m)	3.1m (2.0m - 11.5m)
**Sensitivity analysis–one lifetime test**	**Non-US born**	**2x**	80k (59k - 102k)	25k (11k - 32k)	7.2b (6.6b - 7.8b)	1.4b (1.2b - 1.5b)	12k (5.8k - 16k)	116k (89k - 223k)	80k (62k - 145k)
	**Medical risk factor**	**2x**	93k (74k - 129k)	13k (5k - 19k)	6.8b (6.4b - 7.4b)	0.9b (0.8b - 1.0b)	5.2k (2.2k - 8.3k)	182k (113k - 414k)	142k (90k - 297k)
	**Non-US born + medical risk factor**	**2x**	80k (59k - 106k)	26k (13k - 32k)	7.9b (7.5b - 8.3b)	2.0b (1.8b - 2.1b)	12k (6.2k - 15k)	163k (131k - 303k)	114k (95k - 194k)
	**Universal**	**2x**	80k (64k - 106k)	25k (10k - 34k)	9.8b (9.4b - 10b)	3.9b (3.8b - 4.1b)	13k (5.6k - 17k)	309k (226k - 691k)	216k (163k - 419k)

Simulations of target group testing were run separately and have slightly different basecase results due to stochastic nature of model, explaining minor differences in values. Incremental comparisons are within risk-group set (ie, within non-USB). k = 1,000, m = million, b = billion.

Our sensitivity analysis of a 2-fold increase in once-in-lifetime testing was more cost-effective. The most cost-effective option was a 2-fold increase in TTT of non-USB, which added 11,000 discounted QALYs and had an ICER of $116,000 per QALY gained over the base-case, restricted to individuals who remain in California (Table 3). Individuals who leave the state saved a discounted 4,100 QALYs and $53 million in active disease treatment, improving the overall cost-effectiveness ratio in the 2-fold non-USB scenario to $80,000 per QALY gained versus the base case. Other strategies were above a $100,000 per QALY gained compared to the base-case: $182,000 for those with medical risk factors, $163,000 in the non-USB and those with medical risk factors, and $309,000 in the general population.

## Discussion

Our model predicts slowed progress against the epidemic without substantial investments in targeted LTBI testing and treatment (TTT). A continuation of current TB prevention and care activities will only decrease the number of active cases by an estimated 1.3% per year.

This finding is in keeping with other TB models in low-incidence settings [2] where the epidemiology has shifted away from disease due to recent transmission and toward disease caused by reactivation of LTBI. Without efforts to test and treat the remaining pool of LTBI, the incidence of TB cases may plateau at current levels.

Scale-up of TTT strategies could have substantial impact on TB cases and rates, averting tens of thousands of cases in the next 10–15 years. These findings suggest that reaching pre-elimination, or 10 cases per million population, is potentially within reach. One scenario, a 10-fold increase in TTT of all Californians, meets the pre-elimination goal by its proposed target of 2035. However, this represents testing approximately two-thirds of the population on an annual basis and is likely to be infeasible. The model offered more reasonable paths to reach pre-elimination by 2065. A 10-fold scale-up in all non-USB Californians and those with medical risk factors would require an additional investment of undiscounted $38 billion (four-fold above current spending) between 2017 and 2065. Using our best-estimate simulations, there would be a 92% chance of achieving pre-elimination by 2065 with this strategy. However, short term gains can be made with the TTT strategies we modeled, roughly decreasing the California TB case burden by half in 10 years. supporting the case for stakeholders to promote TTT with existing interventions even as new ones need to be developed.

Our most aggressive TTT option, a 10-fold increase in all Californians, achieved a case rate of 8 (95% UI 4–16) active cases per million by 2050, eight-fold above the elimination goal. Given the 2.6 million individuals with LTBI in California, even successfully treating every individual with 3HP would not lead to prompt elimination but would require additional reductions in LTBI prevalence among new immigrants and transmission over time. Similar results have been found in other models; Hill *et al* found that, on a national level, TB elimination was not possible in the US by 2100.^6^

Despite this, we found great value in targeting high risk groups. TTT in persons who are non-USB or have medical risk factors will achieve pre-elimination for a smaller expenditure than needed for TTT of all Californians. However, none of our main strategies could be cost-effective at a $100,000 cost-per-QALY willingness-to-pay threshold. This is largely because our main strategies allow re-testing once a year. However, restricted to single lifetime testing, would save 11,000 discounted QALYs at $80,000 per QALY gained versus the current TTT level by doubling TTT using QFT tests and 3HP/DOT treatment in the non-USB population. This aligns well with recent estimates of cost effectiveness of combinations of IGRA and 3HP in the non-USB. Tasillo *et al*. studied IGRA and 3HP in the non-USB with no medical risk factors, finding an incremental cost-per-QALY gained of $83,000, in 2015 dollars, compared to no testing [17]. Yeats *et al*. estimated a cost-effectiveness ratio of $58,334, in 2013 dollars, per QALY gained compared to no testing for all non-USB persons, utilizing QFT and four months of rifampin [10].

This model, like all models, has limitations. First, consistent with national guidelines, 3HP was modelled as DOT [18]. A clinical trial of 3HP by self administration is ongoing; early results have shown similar completion rates to 3HP/DOT [19]. Removing direct observation from 3HP therapy would lower the cost from $840 to $520, improving cost-effectiveness ratios [20–21]. Second, we did not account for economies of scale in TTT. A 2- to 10-fold increase in the number of tests being administered might diminish costs. On the other hand, a large scale-up in TTT may require additional start-up expenses (e.g., hiring and training of staff) not accounted for by the model. Third, we used a non-geographic transmission model, instead portraying non-random mixing by nativity and race/ethnicity which are important determinants [22]. Fourth, our use of two phases of reactivation rates (recent vs. remote transmission) is a simplification. Rate of reactivation probably declines over time in a non-linear function [23]. Fifth, because of limitations in sample size, we did not use California-specific estimates of LTBI prevalence, but instead relied on national-level NHANES estimates and adjusted these estimates to better reflect the demographics of California. If risk of LTBI (controlled for race/ethnicity, USB/non-USB, and age) is significantly different in California than nationally, our estimates may be imprecise or biased.

Our model, as of many disease models, poorly accounts for hidden populations, in particular the undocumented and unhoused. In 2015, an estimated 15% of TB cases in California were from individuals with unclear documentation status and 5% were in unhoused individuals [24]. Our demographic data source, the American Community Survey, does not inquire about documentation status or housing, nor does it include institutionalized persons. For this reason, we were unable to specifically model these individuals. Undocumented immigrants may be less likely to seek and complete LTBI therapy, more likely to live in unhealthy housing, and be more at risk for TB from these and other social determinants [25]. Similarly, the unhoused population is particularly at-risk because of intra-population transmission as well as a higher prevalence of risk factors, such as HIV, drug use, and smoking [26–28]. It is difficult to imagine TB elimination occurring without a concerted effort to reach and treat undocumented and unhoused Californians.

Last, the changing demographics of California will affect future TB cases in unpredictable ways. We did not model changes in immigration patterns, instead assuming the next 50 years of immigrants will resemble the recent California immigrants in the 2014 American Community Survey. Evolving national immigration policy and global economic shifts will alter the flow of immigrants, with implications for TB. Similarly, TB control measures in countries of origin for California immigrants will affect TB here.

In summary, our model suggests that scale-up of targeted testing and treatment for LTBI can greatly accelerate California towards TB elimination. New guidelines, such as the California Risk Assessment [29] and the US Preventive Services Task Force Recommendations for LTBI Screening [30] provide guidance and authorization to increase TTT of some populations we have modeled. By increasing testing in the non-USB and those with medical risk factors, significant morbidity and mortality can be averted. However, our model also suggests that a sole strategy of increased LTBI testing and treatment in high-risk populations is unlikely to result in meeting pre-elimination and elimination goals, and that universal, untargeted testing and treatment is unlikely to be cost-effective. These results point to the need for novel diagnostic, therapeutic, or implementation strategies to meet epidemic targets in a cost-effective manner.

## Supporting information

S1 File Technical supplement including table of estimated LTBI prevalence in selected demographic groups (15 or older California population); table of LTBI prevalence over time (15 or older California population); and table of estimates of test performance for modeled diagnostic tests. (DOCX)
