# Supplementary material for: Outlook for tuberculosis elimination in California: An individual-based stochastic model
Source: PLoS One. 2019 Apr 9;14(4):e0214532. doi: 10.1371/journal.pone.0214532 (PMC6456190; doi:10.1371/journal.pone.0214532)
Supplement: S1 File — (DOCX) [file pone.0214532.s001.docx]

**Online Supplement**

**Locally-interacting Markov chains**

The model simulates not only tuberculosis health states, but the states for six medical risk factors in addition to age group, sex, race, length of time in US, and country of birth for each modelled individual. In a traditional Markov compartmental model, this would have generated more than 30 million compartments, requiring a prohibitive 900 million state transition calculations per cycle. This so-called “state space explosion” normally prevents models from developing a significant level of resolution (E1). To avoid this conundrum, a global transition matrix can be partially decomposed into local matrices. If these matrices interact (such as through functional transition probabilities), they are referred to as *locally-interacting Markov chains*.

This is the strategy that we implemented within our model. A chain was created for each phenomenon we modelled (TB, HIV, age group, etc). Individuals occupy a single state in *each* chain. For every cycle, transitions are calculated in a per-person, per-chain approach. Chains are calculated in a random order as to not skew the effect of one chain. To allow for interactions between chains, transition probabilities could be represented as a function of an individual’s states in other chains. This approach has been used previously in the CORE diabetes model (E2).

## Technical specifications

The model was encoded using Python (Python Software Foundation, Beaverton, OR) and Go (Google Inc, Mountain View, CA). Go is a fast, compiled, statically-typed language designed for concurrency, and has been used previously in computational biology (E3). To our knowledge, this is the first published use of Go in public health research. The link to our model programming code is openly accessible (<https://github.com/alexgoodell/california_tb>). Our raw results data set is also currently publicly accessible, at <http://34.201.26.231/limcat/hdd/share>.

**Demographic information**

We modeled only persons 15 years of age or older, who comprise more than 90% of those who develop active TB cases in California (E4). A one-month time step was utilized, and simulations were run from 2001 to 2065. Demographic information was obtained for the State of California for 2001-2014 from the American Community Survey (ACS) via the Integrated Public Use Microdata Series (IPUMS) database (E5). Relevant variables included: sex, race/ethnicity, age, years in the US, migration status, and birthplace. Ethnicities included Asian, black/African-American, Hispanic, non-Hispanic white, and other. Age was grouped by 5-year intervals starting at 15 and capped at 80 or older. Birthplace information was consolidated to include only the United States, Mexico, Central America, and the WHO 22 high burden countries in 2014 (E6). The remaining birthplaces were grouped by their WHO region. The demographics of healthcare workers with direct patient contact were identified using the ACS, and these individuals were a distinct group in the model that was assumed to require yearly testing by their employer.

At the beginning of the simulation, a random sample of 300,000 individuals from California was selected from the 2001 ACS dataset. For each subsequent time step, the cohort was updated with a sample of individuals representing those maturing into the age range and those immigrating to California, as specified by a place of residence outside of California one year ago as reported in the ACS. Individuals exited the model by dying of TB or non-TB causes or emigrating out of California. Death rates were derived from the 2011 US Social Security Actuarial Life Tables (E7). Emigration rates were set through calibration as described below.

For each cycle after 2014, we sampled from the 2014 dataset. We assumed no change in the birthrate or immigration rate from 2014 forward.

**LTBI prevalence**

The NHANES 2011-12 public dataset was used to calculate national TST positive prevalence (induration reading ≥ 10mm) for overall United States, split into US-born and non-US-born with each stratified by race/ethnicity (non-Hispanic white/Hispanic/black/Asian/other) and age (15-24, 25-44, 45-64, 65+) (E8). Although our calibration period began in 2001, the NHANES 2011-12 dataset was chosen because it included more detailed race/ethnicity measurements, and the TST prevalence estimates were not statistically significant between 1999-2001 and 2011-2012 surveys (E9). In our analysis, TST positivity was used as a proxy for LTBI infection. Stratified point estimates were provided by the California Department of Public Health in the form of 2 tables (US-born, non-US-born) with 30 cells (6 age groups × 5 race groups) comprising TST-positive prevalence. Unmodified NHANES-supplied survey weights were used in these calculations, thus supplying us with national-level estimated TST-positive prevalence.

Some of the cell entries in these tables were missing due to lack of data in NHANES. TST-positive prevalence for an empty cell in age group *i* and race group *j* was imputed by multiplying the overall prevalence for that table (US-born or non-US-born) by the product of the ratio of prevalence in age group *i* (from the non-missing cells) to total prevalence for that subpopulation (US-born or non-US-born) and by the ratio of prevalence in race group *j* to total prevalence. Marginal prevalence for age and race groups were calculated from NHANES as were overall US-born or non-US-born prevalence. For example, there were no TST-positive readings for US-born Asians aged 45-64. The prevalence for this group was imputed as *p* × (*p_i_*_+_ /*p*) × (*p*_+_*_j_* /*p*), where *p* is US-born prevalence, *p_i_*_+_ is prevalence for USB age 45-64, and *p*_+_*_j_* is prevalence for US-born Asian. To expand to estimates for 5-year age groups, 15-19, 20-24, …, 75-79, 80+ it was assumed that prevalence within larger age groups was constant across smaller groups. For example, the prevalence calculated for 25-44 was assumed to hold uniformly for 25-29, ..., 40-44. These give prevalence estimates *p_ij_* for age group *i* and race group *j*.

California demographic information concerning age, race, and country of birth were obtained from the ACS in 2001, as our model began its calibration in 2001 (E5). In order to estimate prevalence in California within these country-of-birth groups, previously-published estimates of LTBI infection in country of origin (E10) were adjusted as follows.

First, the non-USB population derived from ACS was grouped by their country of birth, specifically those from Mexico, Central America, and the WHO 22 high burden countries in 2014: Afghanistan, Bangladesh, Brazil, Cambodia, China, Democratic Republic of Congo, Ethiopia, India, Indonesia, Kenya, Myanmar, Nicaragua, Nigeria, Pakistan, Philippines, Russian Federation, South Africa, Thailand, Uganda, United Republic of Tanzania, Viet Nam, and Zimbabwe (E6). The remaining birthplaces were grouped by their WHO region: African Region, Region of the Americas, South-East Asia Region, European Region, Eastern Mediterranean Region, and the Western Pacific Region.

Second, national NHANES-derived non-US-born prevalence by race categories were weighted according to the ACS race distribution in California for each country of birth and summed to produce prevalence estimates of country of birth populations residing in California. Thus, if *w_jk_*^C^ are the ACS-derived race population proportions for country of birth *k* (which sum over race groups *j* to 1), and *p*_+_*_j_* are NHANES-derived non-USB prevalence estimates for race categories *j*, the estimated prevalence in California for country of birth *k* is *p_k_*^N^ = ∑*_j_* *w_jk_*^C^ *p*_+_*_j_* These estimates are consistent with NHANES prevalence by race for non-US-born.

Third, as prevalence among race for California may differ from overall national NHANES estimates, alternate estimates for race groups were calculated based on the prevalence of LTBI in country of birth as reported in Dye. These were combined with the ACS demographic breakdowns of each race group by country of birth to produce race prevalence estimates that are consistent with LTBI prevalence in the country of birth. If *w_jk_*^R^ are the ACS-derived country of birth population proportions for race *j* (which sum over *k* to 1), and *p_k_*^D^ are the Dye prevalence estimates in country of birth *k*, the estimated prevalence in California for race *j* is ∑*_k_* *w_jk_*^R^ *p_k_*^D^.

Fourth, the disagreement between this estimate, which is almost certainly incorrect since LTBI prevalence in non-US countries is known to differ from that among persons in the US originating from those countries, is quantified by its ratio with the corresponding demographically weighted estimate of prevalence in race group *j*. By summing these ratios over race groups *j*, a weighted ratio is obtained which represents the difference in LTBI burden between the sample of immigrants from a nation and the nation itself. The prevalence in California for the population whose country of birth is country *k* is thus estimated, using previous notation, as:

$$p_{k}^{\text{D}}\times\sum_{j} \frac{w_{jk}^{\text{C}} p_{+j}}{\sum_{m} w_{jm}^{\text{R}}p_{m}^{\text{D}}}$$

This is interpretable as the estimate of LTBI prevalence in the country of birth *k* adjusted by California demographics and national-level NHANES race-group estimates.

Fifth, the ratio of this quantity to the earlier estimate for country of birth *k*, that is *p_k_*^N^ = ∑*_j_* *w_jk_*^C^ *p*_+_*_j_* , gives a prevalence ratio PR*_k_* for final adjustment for country of birth *k*. The resulting overall race-group estimates that result from these adjustments are similar to those from NHANES, which lends confidence to their use.

Finally, LTBI prevalence in California for age group *i*, race group *j*, country of birth *k*, and sex *s* (female, male), is estimated as *p_ij_* × PR*_k_* × SR*_s_*, where *p_ij_* and PR*_k_* are defined above and SR*_s_* is the ratio adjustment of NHANES prevalence in non-US-born for females (*s*=0) and males (*s*=1) divided by the average of those two prevalence estimates (Table E1, Table E2).

**TB Chain**

The TB chain had 68 states, representing all the disease states and testing/treatment possibilities. Individuals began in either uninfected, slow latent (remote LTBI), fast latent (recent LTBI), or slow latent status-post six months of isoniazid treatment. LTBI prevalence was estimated from the 2011-2012 NHANES by race/ethnicity, age group, and US-born/non-US-born status. We began the model with 16% of US-born LTBI and 12% of NUSB LTBI previously treated, as suggested by data from NHANES (E11). The percent of LTBI from recent-transmission was 1.8% and 4.2% of all LTBI for US-born and non-US-born, respectively, which was interpolated from recent-transmission cases from 2014 and the trend of pediatric cases from 2001-2014. This process is described fully in the “calibration” section below. LTBI prevalence in NUSB entrants was assumed to decline by a relative 1.9% annually as an approximation of global TB control efforts (E12).

Individuals in slow latent or fast latent could self-clear the infection at a set rate or progress to active disease. Active disease was modeled initially as an untreated group, with a 90% monthly transition into a set of tunnel states representing six months of active disease treatment. Individuals could die or default from active treatment.

**LTBI testing**

Individuals were chosen for testing through an uptake probability. If individuals qualified for a certain testing intervention, they would have a chance to be tested every cycle. Two tests were analyzed: tuberculin skin testing (TST) and use of QuantiFERON-TB Gold (QFT). TST and QFT had total costs of $9.50 and $84.35 respectively, and we assumed a base sensitivity of 83% and 85%, respectively. Specificities of 95% and 99% were used for TST and QFT, respectively. We assumed that the TST was less specific in the non-US-born population due to the Bacillus Calmette–Guérin (BCG) vaccine, and that the immunosuppressive effects of HIV and ESRD had a negative impact on both TST and IGRA test performance (Table E3). Treatment uptake after a positive test varied: 83% was used for QFT, while 76% was used for TST. To evaluate the cost of testing, we included the costs of test reagents, labor, and recruitment. Individuals were able to be re-tested for LTBI at any time within one year, unless they have previously completed treatment.

**LTBI treatment**

LTBI treatment was initiated after being tested positive; both true positives and false positives could undertake treatment. We evaluated two treatment options: self-administered isoniazid for six months (6H) and three months of weekly directly-observed isoniazid and rifapentine (3HP/DOT). We assumed constant monthly drop out rates of 7.4% (5.5 - 9.7) for the isoniazid-only arm and 6.4% (4.8 - 8.0) in the 3HP/DOT arm (E13–15). This results in a 62% (54 – 71) completion for 6H and an 82% (0.62 –1.00) completion for 3HP.

If individuals underwent a full course of treatment, they achieved lifetime protection, were not susceptible to reinfection, and would not undergo future testing or treatment; those dropping out before treatment completion garnered no benefit. The effects of multidrug-resistant TB were not incorporated.

**Transmission**

Each case of active TB was assumed to propagate additional cases of LTBI, ie a *transmissibility coefficient*, with 80% of that risk accounted for within an individual’s race/ethnicity and nativity (US-born/non-US-born) group, and 20% within the general population. This is consistent with evidence that recent transmission occurs more commonly within race/ethnic minority and immigrant populations within California (E16). We included active case finding, assuming that each active case investigation identified 2.5 infections, 75% of which were recent transmissions. These assumptions were estimated from internal CDPH records of case-finding. The overall transmissibility coefficient, as well as coefficients for specific subpopulations, were varied through calibration as described below. The final overall starting coefficient was 4 cases of LTBI for every case of active disease.

**Medical risk factors**

We estimated the prevalence of each risk factor in California using California-specific data if possible, and national-level estimates otherwise (Table E4).

No data source was available for TNF-alpha use, so we estimated its use from the prevalence of the use of these drugs in rheumatoid arthritis and IBD (Table E4). Individuals could have more than one risk factor, and additional relative risks were assumed to be multiplicative. Thus, prevalence of each medical risk factor was stratified by sex, age, race/ethnicity and nativity where available.

To estimate the relative risk (RR) of progression to active disease, we conducted a literature review and arrived at expert consensus among team members as to which estimates were most suitable for the at-risk Californian population (Table E4). Each medical risk factor’s independent risk of mortality was incorporated as a standardized mortality ratio, which yielded a life expectancy once the conditions were distributed within the population.

**Base case and intervention strategies**

We created a basecase scenario that represents current testing and treatment, with healthcare workers required to have annual TB tests (4.6% of the population) were modeled as a distinct group (E5). An additional random 2% of the population received a test every year, as determined through calibration. The base case scenario utilized a 50/50 mixture of the TST and QFT and LTBI therapy of six months of self-administered isoniazid (6H).

We assessed four different strategies applied to the California population: (1) increased TTT in all with medical risk factors regardless of nativity, (2) increased TTT in all non-US-born, (3) increased TTT in all non-US-born and all with medical risk factors regardless of nativity, and (4) increased TTT in all Californians (universal). For each strategy, we estimated the effects of increasing TTT by a factor of 2, 4, or 10. This created a total of twelve simulations in addition to the base case. Additional testing utilized QFT and treatment was 3HP/DOT. For each of these strategies, we evaluated the total cases and net costs from 2017 to 2065. Future costs and QALYs were discounted 3% (1-5%) per year (E17).

**Calibration**

To calibrate the model, we employed a step-wise, non-computational approach which captured the historical pattern of active TB from 2001 onward stratified by sex, nativity, race/ethnicity, and medical risk factors.

First, we calibrated the prevalence of each MRF to match known trends in their prevalence from 2001 to 2014 by adjusting the initial prevalence and/or incidence to match established trends derived from available data. For example, initial HIV prevalence was set at the reported HIV prevalence in 2001. To match the estimated prevalence of HIV from 2002 to 2014, the incidence rate was adjusted until prevalence aligned with historical trends. The comorbidities come with a higher risk of death, and individuals with these conditions were given a higher yearly chance of death (standardized mortality rate) in line with the literature. These values were not adjusted during calibration. As TNF-alpha inhibitor usage was not available for California, TNF-alpha inhibitor usage was calibrated to fall within the 95% confidence interval of TNF-alpha usage in four waves of NHANES data reported by Yeats et al. (10).

Second, adjustments were made to race- and nativity-specific risks of TB reactivation to match reported cases in 2001 (Table 1). US-born Asians, Blacks, Hispanics, Whites, and individuals of other ethnicities required a 1.2, 2.2, 1.5, 2.0 and 0.5 relative increase in risk of progression rates, respectively. Non-USB Asians, Blacks, Hispanics, Whites, and individuals of other ethnicities required a 1.48, 1.5, 0.83, 0.5, and 2.0 relative adjustment. This is straightforward, and speaks to the relative risks not captured by our comorbidities. The USB/NUSB-race designation was used as we felt it best captured these unknown risks. We included a NUSB diabetes-specific adjustment due to an increased risk in this group, as noted in other research (63).

Third, USB whites required an additional 6% yearly reduction in risk of progression to match data from 2002 to 2014.

Fourth, we adjusted the relative risks of reactivation associated with each risk factor to match the reported number of cases in those with a medical risk factor (Table E4). Table E4 shows the pre-calibration and post-calibration values. Only two were modified, and both were within reported literature values.

Fifth, we adjusted the number of so-called imported cases (active cases within one year of entry into the US). Between the 2001 to 2014 time-period used for calibration, there was a significant drop in adult TB cases, some of which may be attributable to a decline in active disease importation (64). In 2001, there were 444 such cases (14% of adult cases) within one year of entry. By 2014, there were only 160 (8% of adult cases). To account for this change, we reduced the incidence of these cases by 5% per year to match the historical trend, and continued the trend beyond 2014.

Six, we adjusted our transmission coefficient to match the number of cases due to recent (within three years) transmission. In 2014, 83 cases in the USB and 143 in the NUSB were estimated to be from recent transmission within the US, representing 22% of USB, 8.5% of NUSB, and 11% of total adult cases (24). Since recent transmission is estimated through genetic analysis, no past estimates for what proportion of active cases were due to RT is available. Estimation is possible. One useful marker for transmission is the number of cases found in the population under 5 years of age (U5), as these are by-definition transmissions occurring within the last 5 years (65). In 2014, 55 cases (2.6% of all cases) were those found in the U5 population, compared to 134 (4%) in 2001. This represents a 59% reduction in the number of U5 cases per year. If we assume that U5 cases are a valid surrogate marker for transmission, we can use this proportion to estimate the level of transmission in 2001. We build a simple linear model to describe the relationship between year and risk of recent-within-US using under-five cases and adult cases as our input data, stratified by USB/NUSB. Using this method, we estimated that 551 adult cases in 2001 were due to recent transmission. Then to match the downward trend of cases due to recent transmission, the transmission coefficient was reduced by 2.5% per year starting in 2001 for the USB and 1% per year for the NUSB.

**Sensitivity Analysis**

Along with our sensitivity analysis showing the effects of one-time testing, we completed a probabilistic sensitivity analysis using a standard Monte Carlo simulation that sampled variables from a triangular distribution using the ranges described above (E22). The followed variables were varied:

*- Drop out rate from LTBI therapy*

*- Drop out rate from active TB therapy*

*- Risk of progression from recent*

*- Risk of progression from remote infection*

*- Starting prevalences of non-demographic risk factors (DM, HIV, ESRD, SOT, Smoking)*

*- Transition probabilities in and out of non-demographic risk factors (DM, HIV, ESRD, SOT, Smoking)*

*- Proportion of individuals that enroll in treatment after a positive TST LTBI test*

*- Proportion of individuals that enroll in treatment after a positive QFT/TSPOT test*

*- Number of LTBI cases caused by one active case*

*- Number of secondary TB cases caused by one active case*

*- Efficacy of therapies*

*- Proportion of LTBI treated starting simulation*

*- Proportion of started who complete treatment for each therapy*

*- Total cost of LTBI therapy per month by modality*

*- Total cost of LTBI testing per month by modality*

*- Active disease cost per month*

*- TB-related mortality per month*

*- QALYs gained averting one case of active TB*

*- Discounting*

*- All "multiplicative coefficients" (ie adjusting risk of progression by race-nativity)*

All scenarios were executed in parallel sets using the same sampled values for input parameters values and the random number sequence that determines events in the simulation. Individual simulations were compared to the simulations within that sampling set to produce estimates such as cost or utility difference. The sets were repeated 250 times.

The Go Godes package was used for this process (E23).

**Year 1 Budget analysis**

Costs for Year 1, by type of cost and intervention strategy, are reported in Table E5.

## Tables

**S1 Table.** Estimated LTBI prevalence in selected demographic groups (15 or older California population).

|  | **US born** | **Non-US-born** |
| --- | --- | --- |
| **Total** | 2.4% | 19.4% |
|  |  |  |
| **Sex** |  |  |
| Male | 3.1% | 24.4% |
| Female | 1.7% | 14.8% |
|  |  |  |
| **Race/Ethnicity** | |  |
| Asian | 5.1% | 30.1% |
| Hispanic | 2.7% | 13.8% |
| Black | 7.1% | 21.7% |
| White | 1.4% | 15.1% |
| Other | 2.2% | 25.5% |
|  |  |  |
| **Age** |  |  |
| Age 15-19 | 0.4% | 11.1% |
| Age 20-24 | 0.7% | 12.5% |
| Age 25-29 | 3.3% | 19.6% |
| Age 30-34 | 3.2% | 18.4% |
| Age 35-39 | 2.9% | 20.2% |
| Age 40-44 | 2.1% | 18.6% |
| Age 45-49 | 4.4% | 23.1% |
| Age 50-54 | 3.6% | 26.1% |
| Age 55-59 | 3.7% | 24.2% |
| Age 60-64 | 3.7% | 27.3% |
| Age 65-69 | 1.7% | 13.4% |
| Age 70-74 | 0.5% | 9.4% |
| Age 75-79 | 0.8% | 13.0% |
| Age 80+ | 1.5% | 9.1% |

**S2. Table.** LTBI prevalence over time (15 or older California population).

|  | **2001** | **2014** | **2030** | **2040** | **2050** |
| --- | --- | --- | --- | --- | --- |
| USB | 2.1% (1.6 - 2.6%) | 1.6% (1.1 - 2.1%) | 1.3% (0.9 - 1.8%) | 1.2% (0.9 - 1.6%) | 1.2% (0.9 - 1.5%) |
| Non-US-born | 21.6% (19 - 25%) | 17.1% (16 - 20%) | 13.2% (11 - 16%) | 11.4% (11 - 13%) | 9.9% (9.5 - 12%) |

**S3 Table.** Estimates of test performance

|  |  | ***Sensitivity*** |  |  | ***Specificity*** |  |  |  |
| --- | --- | --- | --- | --- | --- | --- | --- | --- |
| ***Name*** | ***Base*** | ***Low*** | ***High*** | ***Reference*** | ***Base*** | ***Low*** | ***High*** | ***Reference*** |
| USB TST | 0.83 | 0.71 | 0.87 | (E24–37) | 0.95 | 0.82 | 0.99 | (E24–37) |
| USB QFT | 0.85 | 0.70 | 0.97 | (E24–37) | 0.99 | 0.98 | 1.00 | (E24–37) |
| USB TSPOT | 0.85 | 0.70 | 0.97 | (E24–37) | 0.98 | 0.97 | 1.00 | (E24–37) |
| NUSB TST | 0.83 | 0.71 | 0.87 | (E24–37) | 0.82 | 0.47 | 0.92 | (E24–37) |
| NUSB QFT | 0.85 | 0.70 | 0.97 | (E24–37) | 0.99 | 0.98 | 1.00 | (E24–37) |
| NUSB TSPOT | 0.85 | 0.70 | 0.97 | (E24–37) | 0.98 | 0.97 | 1.00 | (E24–37) |
| HIV TST | 0.75 | 0.68 | 0.83 | (E38) | 0.64 | 0.58 | 0.70 | *Assumed* |
| HIV QFT | 0.59 | 0.53 | 0.65 | (E38) | 0.89 | 0.80 | 0.98 | (E38) |
| HIV TSPOT | 0.59 | 0.53 | 0.65 | Assumed | 0.89 | 0.80 | 0.98 | Assumed |
| ESRD TST | 0.51 | 0.46 | 0.56 | (E39) | 0.64 | 0.58 | 0.70 | (E39) |
| ESRD QFT | 0.53 | 0.48 | 0.58 | (E39) | 0.69 | 0.62 | 0.76 | (E39) |
| ESRD TSPOT | 0.53 | 0.48 | 0.58 | Assumed | 0.69 | 0.62 | 0.76 | Assumed |

**S4 Table.** Relative risk (RR) for progression to active disease, prevalence, 2014 cases in individuals with those risk factors, and life expectancies for medical risk factors

| **Risk group** | **Stratified by** | **Modeled prevalence (%), 2014** | **Cases in 2014 (%)** | **Reference for stratified prevalence estimate** | **Calibrated trends 2001-2011 using** | **RR progression (pre- calibration)** | **RR progression (post-calibration)** | **Reference for RR** |
| --- | --- | --- | --- | --- | --- | --- | --- | --- |
| Diabetes | Age, sex, race/ethnicity, nativity | 8.9 | 25 | (E40) | (E41) | 1.6  (1.3-3.60) | 1.6 | (E42) |
| Smoking | Age, sex, race/ethnicity, nativity | 12.7 | 13 | (E40) | (E41) | 2.5  (1-4) | 2.5 | (E43) |
| HIV | Age, sex, race/ethnicity | 0.41 | 4.2 | Unpublished data, CA Department of AIDS | (E44) | 12  (2.9-22) | 5.4 | (E45) |
| TNF-alpha | Age, sex | 0.80 | 0.98 | Expert opinion, Derived from (E46–51) | Constant as a proportion of the population (E18) | 4.7  (2.5-5.3) | 4.7 | (E18) |
| Solid-organ transplants | Age, sex, | 0.17 | 1.1 | (E52) | Constant as a proportion of the population | 2.4  (1.7-18) | 2.4 | (E53) |
| ESRD | Diabetes, age, sex, race/ethnicity | 0.30 | 4 | (E54) | *(E54)* | 11  (2-20) | 10.4 | (E55) |

**S5 Table.** Budget analysis, Year 1

**References**

1. Plateau B, Atif K. Stochastic automata network of modeling parallel systems. *Softw Eng IEEE Trans On* 1991;17:1093–1108.

2. Palmer AJ, Roze S, Valentine WJ, Minshall ME, Foos V, Lurati FM, Lammert M, Spinas GA. The CORE Diabetes Model: Projecting Long-term Clinical Outcomes, Costs and Costeffectiveness of Interventions in Diabetes Mellitus (Types 1 and 2) to Support Clinical and Reimbursement Decision-making. *Curr Med Res Opin* 2004;20:S5–S26.

3. Liu W, Islamaj Dogan R, Kwon D, Marques H, Rinaldi F, Wilbur WJ, Comeau DC. BioC implementations in Go, Perl, Python and Ruby. *Database J Biol Databases Curation* 2014;2014.:

4. California Department of Public Health. *Reports of Verified Cases of Tuberculosis Database (S6-RVCT database)*. State of California; 2016.

5. Ruggles S, Genadek K, Goeken R, Grover J, Sobek M. *Integrated Public Use Microdata Series: Version 7.0 [dataset]*. Minneapolis: University of Minnesota; 2017. at <https://doi.org/10.18128/D010.V7.0>.

6. WHO. *Global Tuberculosis Report 2014*. World Health Organization; 2014.

7. Social Security Administration. *Actuarial Life Table. Washington, DC: Social Security Administration*. Baltimore, MD: Social Security Administration; 2015.

8. Centers for Disease Control and Prevention, National Center for Health Statistics (NCHS). *National Health and Nutrition Examination Survey Data*. Hyattsville, MD: US Department of Health and Human Services; 2012. at <https://www.cdc.gov/nchs/nhanes/index.htm>.

9. Miramontes R, Hill AN, Woodruff RSY, Lambert LA, Navin TR, Castro KG, LoBue PA. Tuberculosis Infection in the United States: Prevalence Estimates from the National Health and Nutrition Examination Survey, 2011-2012. *PloS One* 2015;10:e0140881.

10. Dye C, Scheele S, Dolin P, Pathania V, Raviglione MC, others. Global burden of tuberculosis: estimated incidence, prevalence, and mortality by country. *Jama* 1999;282:677–686.

11. Bennett DE, Courval JM, Onorato I, Agerton T, Gibson JD, Lambert L, McQuillan GM, Lewis B, Navin TR, Castro KG. Prevalence of tuberculosis infection in the United States population: the national health and nutrition examination survey, 1999–2000. *Am J Respir Crit Care Med* 2008;177:348–355.

12. Dye C, Lönnroth K, Jaramillo E, Williams B, Raviglione M. Trends in tuberculosis incidence and their determinants in 134 countries. *Bull World Health Organ* 2009;87:683–691.

13. Horsburgh CR, Goldberg S, Bethel J, Chen S, Colson PW, Hirsch-Moverman Y, Hughes S, Shrestha-Kuwahara R, Sterling TR, Wall K, others. Latent TB infection treatment acceptance and completion in the United States and Canada. *CHEST J* 2010;137:401–409.

14. Menzies D, Long R, Trajman A, Dion M-J, Yang J, Al Jahdali H, Memish Z, Khan K, Gardam M, Hoeppner V, others. Adverse events with 4 months of rifampin therapy or 9 months of isoniazid therapy for latent tuberculosis infection: a randomized trial. *Ann Intern Med* 2008;149:689–697.

15. Sterling TR, Villarino ME, Borisov AS, Shang N, Gordin F, Bliven-Sizemore E, Hackman J, Hamilton CD, Menzies D, Kerrigan A, others. Three months of rifapentine and isoniazid for latent tuberculosis infection. *N Engl J Med* 2011;365:2155–2166.

16. Myers WP, Westenhouse JL, Flood J, Riley LW. An ecological study of tuberculosis transmission in California. *Am J Public Health* 2006;96:685.

17. Siegel JE, Weinstein MC, Russell LB, Gold MR. Recommendations for reporting cost-effectiveness analyses. *Jama* 1996;276:1339–1341.

18. Yeats J, Baker, Brian, Basurto-Davila, Ricardo. Priorities for Targeted Testing for Latent Tuberculosis Infection among Foreign‐Born Adults in the United States. 2015;at <http://www.rand.org/pubs/rgs_dissertations/RGSD356.html>.

19. Demlow SE, Oh P, Barry PM. Increased risk of tuberculosis among foreign-born persons with diabetes in California, 2010–2012. *BMC Public Health* 2015;15:263.

20. Centers for Disease Control and Prevention, others. Decrease in reported tuberculosis cases-United States, 2009. *MMWR Morb Mortal Wkly Rep* 2010;59:289.

21. Dodd PJ, Gardiner E, Coghlan R, Seddon JA. Burden of childhood tuberculosis in 22 high-burden countries: a mathematical modelling study. *Lancet Glob Health* 2014;2:e453–e459.

22. Stewart WJ. *Introduction to the numerical solution of Markov chains*. Princeton University Press Princeton; 1994.

23. Goussiatiner A. Godes: Open Source Library to Build Discrete Event Simulation Models in Go/Golang. 2018;at <https://github.com/agoussia/godes>.

24. Wang L, Turner M, Elwood R, Schulzer M, FitzGerald J. A meta-analysis of the effect of Bacille Calmette Guerin vaccination on tuberculin skin test measurements. *Thorax* 2002;57:804–809.

25. Chee CB, Gan SH, KhinMar KW, Barkham TM, Koh CK, Liang S, Wang YT. Comparison of sensitivities of two commercial gamma interferon release assays for pulmonary tuberculosis. *J Clin Microbiol* 2008;46:1935–1940.

26. Harada N, Higuchi K, Yoshiyama T, Kawabe Y, Fujita A, Sasaki Y, Horiba M, Mitarai S, Yonemaru M, Ogata H, others. Comparison of the sensitivity and specificity of two whole blood interferon-gamma assays for M. tuberculosis infection. *J Infect* 2008;56:348–353.

27. Lee J, Choi H, Park I, Hong S, Oh Y, Lim C, Lee S, Koh Y, Kim W, Kim D, others. Comparison of two commercial interferon-γ assays for diagnosing Mycobacterium tuberculosis infection. *Eur Respir J* 2006;28:24–30.

28. Aichelburg MC, Rieger A, Breitenecker F, Pfistershammer K, Tittes J, Eltz S, Aichelburg AC, Stingl G, Makristathis A, Kohrgruber N. Detection and prediction of active tuberculosis disease by a whole-blood interferon-γ release assay in HIV-1–infected individuals. *Clin Infect Dis* 2009;48:954–962.

29. Ruhwald M, Bodmer T, Maier C, Jepsen M, Haaland M, Eugen-Olsen J, Ravn P, others. Evaluating the potential of IP-10 and MCP-2 as biomarkers for the diagnosis of tuberculosis. *Eur Respir J* 2008;32:1607–1615.

30. Palazzo R, Spensieri F, Massari M, Fedele G, Frasca L, Carrara S, Goletti D, Ausiello CM. In-house bulk and single cell antigen-specific whole blood Interferon-gamma assays for surveillance of M. tuberculosis infection. *Clin Vaccine Immunol* 2007;

31. Oxlade O, Schwartzman K, Menzies D. Interferon-gamma release assays and TB screening in high-income countries: a cost-effectiveness analysis. *Int J Tuberc Lung Dis* 2007;11:16–26.

32. Diel R, Goletti D, Ferrara G, Bothamley G, Cirillo D, Kampmann B, Lange C, Losi M, Markova R, Migliori GB, Nienhaus A, Ruhwald M, Wagner D, Zellweger JP, Huitric E, Sandgren A, Manissero D. Interferon-γ release assays for the diagnosis of latent Mycobacterium tuberculosis infection: a systematic review and meta-analysis. *Eur Respir J* 2011;37:88–99.

33. Detjen A, Keil T, Roll S, Hauer B, Mauch H, Wahn U, Magdorf K. Interferon-γ release assays improve the diagnosis of tuberculosis and nontuberculous mycobacterial disease in children in a country with a low incidence of tuberculosis. *Clin Infect Dis* 2007;45:322–328.

34. Bartu V, Havelkova M, Kopecka E. QuantiFERON®-TB Gold in the diagnosis of active tuberculosis. *J Int Med Res* 2008;36:434–437.

35. Tsiouris SJ, Coetzee D, Toro PL, Austin J, Stein Z, El-Sadr W. Sensitivity analysis and potential uses of a novel gamma interferon release assay for diagnosis of tuberculosis. *J Clin Microbiol* 2006;44:2844–2850.

36. Pai M, Zwerling A, Menzies D. Systematic review: T-cell–based assays for the diagnosis of latent tuberculosis infection: an update. *Ann Intern Med* 2008;149:177–184.

37. Mazurek GH, Jereb JA, Vernon A, LoBue P, Goldberg S, Castro KG, Committee IE, Control C for D, (CDC) P, others. Updated guidelines for using interferon gamma release assays to detect Mycobacterium tuberculosis infection, United States, 2010. 2010;

38. Santin M, Munoz L, Rigau D. Interferon-γ release assays for the diagnosis of tuberculosis and tuberculosis infection in HIV-infected adults: a systematic review and meta-analysis. *PloS One* 2012;7:e32482.

39. Sester U, Junker H, Hodapp T, Schütz A, Thiele B, Meyerhans A, Köhler H, Sester M. Improved efficiency in detecting cellular immunity towards M. tuberculosis in patients receiving immunosuppressive drug therapy. *Nephrol Dial Transplant* 2006;21:3258–3268.

40. California Health Interveiw Survey. *CHIS 2001, 2003, 2007, 2011, 2014 Adult Public Use File. [computer file].* Los Angeles, CA: UCLA Center for Health Policy Research; 2015.

41. Centers for Disease Control and Prevention. *Behavioral Risk Factor Surveillance System Survey Data*. Atlanta, GA: Department of Health and Human Services; 2001. at <https://www.cdc.gov/brfss/>.

42. Dobler CC, Flack JR, Marks GB. Risk of tuberculosis among people with diabetes mellitus: an Australian nationwide cohort study. *BMJ Open* 2012;2:e000666.

43. Smith GS, Van Den Eeden SK, Baxter R, Shan J, Van Rie A, Herring AH, Richardson DB, Emch M, Gammon MD. Cigarette smoking and pulmonary tuberculosis in northern California. *J Epidemiol Community Health* 2015;69:568–573.

44. Hall HI, An Q, Tang T, Song R, Chen M, Green T, Kang J. Prevalence of diagnosed and undiagnosed HIV infection-United States, 2008–2012. *MMWR Morb Mortal Wkly Rep* 2015;64:657–62.

45. Zolopa AR, Hahn JA, Gorter R, Miranda J, Wlodarczyk D, Peterson J, Pilote L, Moss AR. HIV and tuberculosis infection in San Francisco’s homeless adults: prevalence and risk factors in a representative sample. *Jama* 1994;272:455–461.

46. Kappelman MD, Rifas–Shiman SL, Kleinman K, Ollendorf D, Bousvaros A, Grand RJ, Finkelstein JA. The prevalence and geographic distribution of Crohn’s disease and ulcerative colitis in the United States. *Clin Gastroenterol Hepatol* 2007;5:1424–1429.

47. Kappelman MD, Rifas–Shiman SL, Porter CQ, Ollendorf DA, Sandler RS, Galanko JA, Finkelstein JA. Direct health care costs of Crohn’s disease and ulcerative colitis in US children and adults. *Gastroenterology* 2008;135:1907–1913.

48. Karve S, Candrilli S, Kappelman MD, Tolleson-Rinehart S, Tennis P, Andrews E. Healthcare utilization and comorbidity burden among children and young adults in the United States with systemic lupus erythematosus or inflammatory bowel disease. *J Pediatr* 2012;161:662–670.

49. Zhang J, Xie F, Delzell E, Chen L, Kilgore ML, Yun H, Saag KG, Lewis JD, Curtis JR. Trends in the use of biologic agents among rheumatoid arthritis patients enrolled in the US Medicare program. *Arthritis Care Res* 2013;65:1743–1751.

50. Rasch EK, Hirsch R, Paulose-Ram R, Hochberg MC. Prevalence of rheumatoid arthritis in persons 60 years of age and older in the United States: effect of different methods of case classification. *Arthritis Rheum* 2003;48:917–926.

51. Cross M, Smith E, Hoy D, Carmona L, Wolfe F, Vos T, Williams B, Gabriel S, Lassere M, Johns N, others. The global burden of rheumatoid arthritis: estimates from the Global Burden of Disease 2010 study. *Ann Rheum Dis* 2014;73:1316–1322.

52. US Department of Health and Human Services. Organ procurement and transplantation network. *Organ Procure Transplant Netw* 2014;

53. Chen C-H, Lian J-D, Cheng C-H, Wu M-J, Lee W-C, Shu K-H. Mycobacterium tuberculosis infection following renal transplantation in Taiwan. *Transpl Infect Dis* 2006;8:148–156.

54. United States Renal Data System. Online Query System. 2015;at <https://www.usrds.org/>.

55. Ahmed A, Karter A. Tuberculosis in California dialysis patients. *Int J Tuberc Lung Dis* 2004;8:341–345.
